# Acetate attenuates perioperative neurocognitive disorders in aged mice

**DOI:** 10.18632/aging.102856

**Published:** 2020-02-26

**Authors:** Cen Wen, Tao Xie, Ke Pan, Yu Deng, Zhijia Zhao, Na Li, Jinjun Bian, Xiaoming Deng, Yanping Zha

**Affiliations:** 1 Faculty of Anesthesiology, Changhai Hospital, Navy Medical University, Shanghai 200433, China

**Keywords:** perioperative neurocognitive disorders (PNDs), neuroinflammation, microglia, acetate, G protein-coupled receptors (GPCRs)

## Abstract

Perioperative neurocognitive disorders are common in elderly patients who have undergone surgical procedures. Neuroinflammation induced by microglial activation is a hallmark of these neurological disorders. Acetate can suppress inflammation in the context of inflammatory diseases. We employed an exploratory laparotomy model with isoflurane anesthesia to study the effects of acetate on perioperative neurocognitive disorders in aged mice. Neurocognitive function was assessed with open-field tests and Morris water maze tests 3 or 7 days post-surgery. Acetate ameliorated the surgery-induced cognitive deficits of aged mice and inhibited the activation of IBA-1, a marker of microglial activity. Acetate also reduced expression of inflammatory proteins (tumor necrosis factor-α, interleukin-1β and interleukin-6), oxidative stress factors (NADPH oxidase 2, inducible nitric oxide synthase and reactive oxygen species), and signaling molecules (nuclear factor kappa B and mitogen-activated protein kinase) in the hippocampus. BV2 microglial cells were used to verify the anti-inflammatory effects of acetate *in vitro*. Acetate suppressed inflammation in lipopolysaccharide-treated BV2 microglial cells, but not when GPR43 was silenced. These results suggest that acetate may bind to GPR43, thereby inhibiting microglial activity, suppressing neuroinflammation, and preventing memory deficits. This makes acetate is a promising therapeutic for surgery-induced neurocognitive disorders and neuroinflammation.

## INTRODUCTION

Perioperative neurocognitive disorders (PNDs) are very common cognitive impairments in older patients who have undergone surgery with anesthesia [1]. The incidence of PNDs varies from 41-75% at seven days to 18-45% at three months post-surgery. PNDs are associated with poor functional recovery and increased mortality after major surgeries [2, 3]. Surgery can trigger acute systemic inflammation, followed by neuroinflammation and synaptic dysfunction, which can lead to hippocampus-dependent cognitive deficits [4–6]. Although pathological events have been reported to be relevant to PNDs [7, 8], there are no effective clinical strategies to prevent or treat PNDs.

Microglia, the resident macrophage-like cells in the central nervous system (CNS), are key contributors to the development of PNDs [9]. Microglia are normally in a resting state, but are rapidly activated by exogenous antigens such as bacteria or viruses, and become neurotoxic when they are overactivated [10]. On the one hand, the activation of microglia may induce the production of inflammatory proteins such as tumor necrosis factor-α (TNF-α), interleukin-1β (IL-1β) and interleukin-6 (IL-6) via the nuclear factor kappa B (NF-κB) and mitogen-activated protein kinase (MAPK) signaling pathways, resulting in neuroinflammation and cognitive deficits [11, 12]. On the other hand, microglial activation may promote the production of free radicals such as reactive oxygen species (ROS, mainly derived from NADPH oxidase 2 [NOX2]) and reactive nitrogen species (RNS). Neurons are susceptible to these active substances, which contribute to oxidative stress and impair neurocognitive function [13, 14]. Therefore, the targeted inhibition of microglial activity may improve neurocognitive function by suppressing neuroinflammation and oxidative stress.

Short-chain fatty acids (SCFAs) are important metabolic products that are mainly produced through the fermentation of dietary fiber and the deacetylation of histones [15]. SCFA metabolites can alter an individual’s immune phenotype and inhibit a variety of signaling pathways in immune cells [16, 17]. Acetate, one such SCFA, has been reported to participate in energy delivery, inflammation and microbial diversification [17], to reduce lipopolysaccharide (LPS)-induced nitric oxide production and to exert antioxidant activity in rat primary astrocytes [18]. Acetate has also been found to inhibit inflammatory responses in different models, such as skin inflammation and colitis models [19, 20]. However, the effects of acetate on PNDs are unclear.

Herein, we investigated whether acetate could suppress microglial activity and improve hippocampus-dependent neurocognitive outcomes in a murine laparotomy model. We then examined whether the effects of acetate depended on G protein-coupled receptor 43 (GPR43) in an *in vitro* model of neuroinflammation. Our study has provided new evidence for acetate as a potential therapeutic for PNDs.

## RESULTS

### Acetate reduced hippocampus-dependent cognitive impairment after surgery

**Figure 1:**
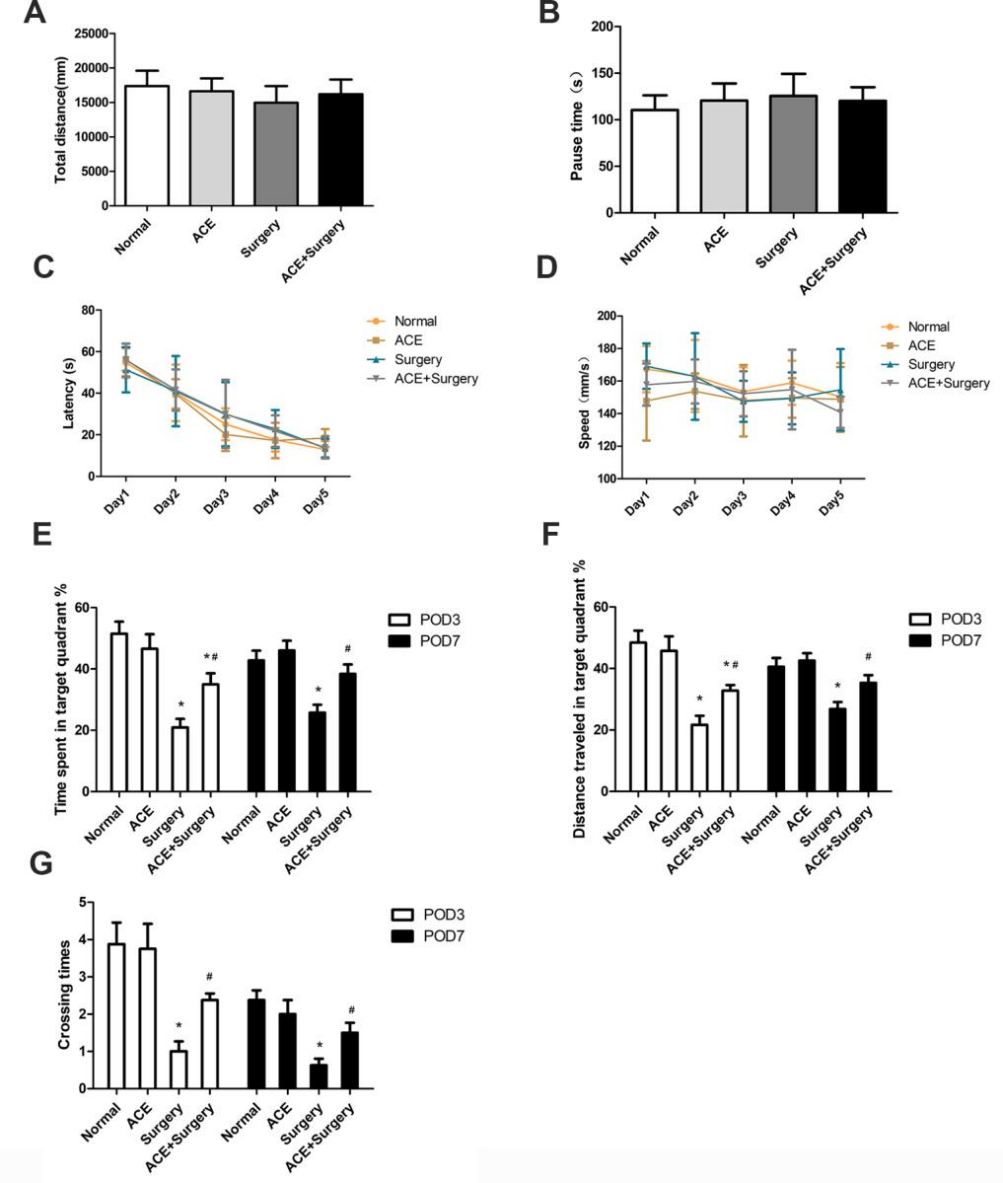
**Acetate improved hippocampus-dependent neurocognition after surgery (n=8).** OFTs were conducted on POD 3. (**A**) Total distance in the OFT. (**B**) Pause time in the OFT. Graphs display the latency (**C**) and average speed (**D**) in the training phase of the MWM test. On POD 3 and POD 7, the percentage of time spent in the target quadrant (**E**), the distance traveled in the target quadrant (**F**) and the crossing times (**G**) in the MWM test were recorded. Data are expressed as the mean±SEM, *P<0.05 vs. the normal and acetate groups, #P<0.05 vs. the surgery group. ACE: acetate.

To determine the effects of acetate on surgery-induced cognitive impairment, we examined four groups of aged mice: a normal group, an acetate-treated group, a surgery group and an acetate-pretreated surgery group (acetate + surgery group). The latter two groups underwent exploratory laparotomies under isoflurane anesthesia, while the former two groups underwent neither surgery nor anesthesia. We assessed the locomotor activity in these four groups of mice through open-field tests (OFTs) on postoperative day 3 (POD 3). After surgery, there were no significant differences among the four groups in the OFT results, including the total distance and the pause time (Figure 1A and 1B), suggesting that the surgery did not cause spontaneous locomotor activity decline.

Then, we evaluated hippocampus-dependent memory performance via the Morris water maze (MWM). In the training phase, no significant differences were found among the groups in latency and average speed (Figure 1C and 1D), indicating that all the mice had learned to find the hidden platform after five days of training. In the probe trial, the percentage of time spent in the target quadrant, the distance traveled in the target quadrant and the number of platform crossings were all notably lower in the surgery group than in the normal group on POD 3 and POD 7, signifying that the surgery had evoked cognitive deficits (which were not due to spontaneous locomotor activity decline, as evidenced by the OFT results). These indicators were greater in the acetate + surgery group than in the surgery group on both POD 3 and POD 7 (Figure 1E–1G), indicating that acetate may have prevented surgery-induced cognitive impairment.

### Acetate attenuated surgery-induced systemic inflammation and neuroinflammation

**Figure 2:**
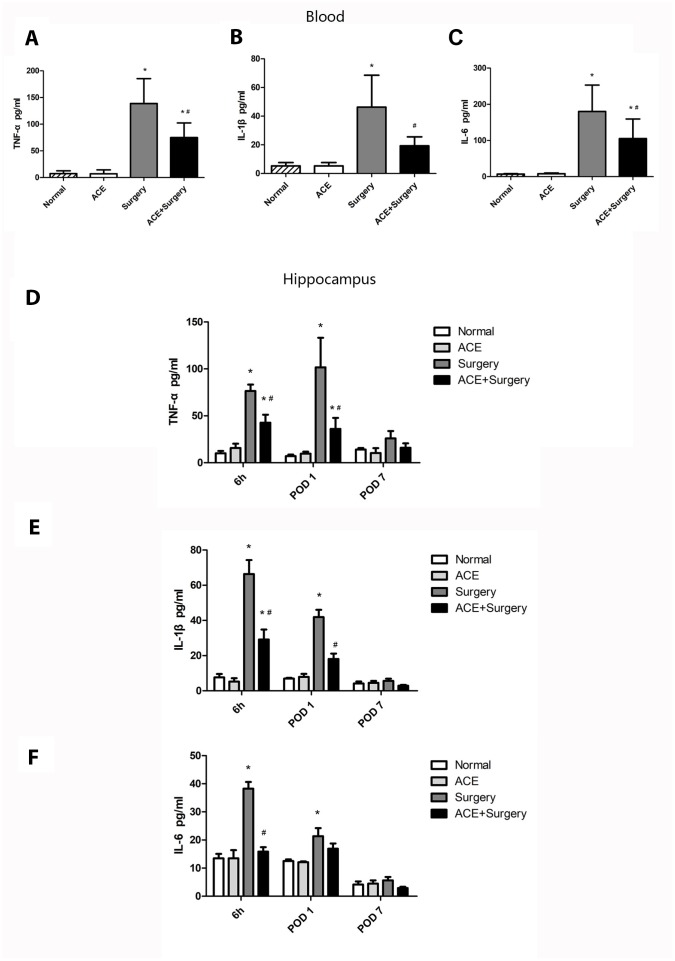
**The effects of acetate on systemic inflammation and neuroinflammation after surgery (n=5-8).** The protein levels of TNF-α (**A**), IL-1β (**B**) and IL-6 (**C**) in the systemic circulation 6 h post-surgery. Graphs display the expression of TNF-α (**D**), IL-1β (**E**) and IL-6 (**F**) in the hippocampus at different time points. Data are expressed as the mean±SEM, *P<0.05 vs. the normal and acetate groups, #P<0.05 vs. the surgery group. ACE: acetate.

We then assessed the acute inflammatory response in the plasma and the hippocampus after surgery. The peripheral blood levels of TNF-α, IL-1β and IL-6 were upregulated 6 h post-surgery, but acetate markedly reduced the levels of these inflammatory proteins (Figure 2A–2C). Similar results were observed for neuroinflammation: acetate dramatically downregulated inflammatory factors in the hippocampus 6 h post-surgery. On POD 1, hippocampal inflammatory cytokine levels were significantly greater in the surgery group than in the normal group. Acetate treatment attenuated the hippocampal expression of IL-1β and TNF-α, but not IL-6, on POD 1 (Figure 2D–2F). On POD 7, cytokine expression did not differ significantly between the surgery group and the normal or acetate groups, suggesting that the inflammation had diminished over time. These results indicated that acetate pretreatment may have suppressed the systemic inflammation and neuroinflammation induced by surgery.

### Acetate treatment inhibited inflammatory signaling pathway activation and oxidative stress in the hippocampus

**Figure 3:**
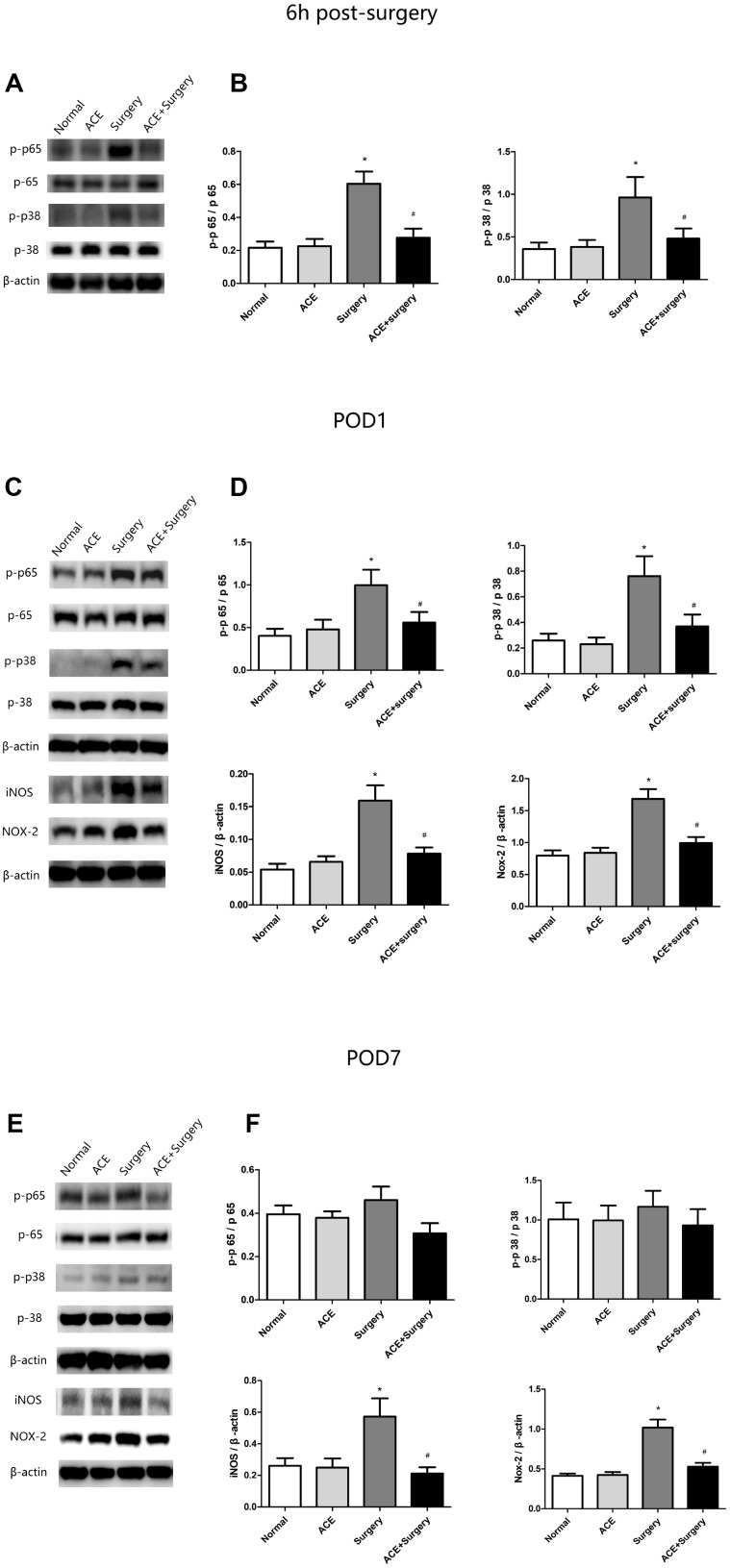
**Changes in inflammatory signaling pathways and oxidative stress markers (n=8).** (**A**) Representative bands display p-p65 and p-p38 protein levels 6 h post-surgery. (**B**) Bar graphs depict the relative quantification of p-p65/p65 and p-p38/p38. (**C**) Representative bands display p-p65, p-p38, iNOS and NOX2 protein levels on POD 1. (**D**) Bar graphs depict the relative quantification of p-p65/p65, p-p38/p38, iNOS/β-actin and NOX2/β-actin on POD 1. (**E**) Representative bands display the protein levels of the above indicators on POD 7. (**F**) Bar graphs depict the relative quantification of these indicators on POD 7. Data are expressed as the mean±SEM, *P<0.05 vs. the normal and acetate groups, #P<0.05 vs. the surgery group. ACE: acetate.

Inflammatory signaling pathways such as the NF-κB and MAPK p38 pathways are vital contributors to multiple inflammatory diseases. The NF-κB and MAPK p38 pathways are activated during the occurrence and development of PNDs, and these pathways induce IL-1β, TNF-α and IL-6 expression, thus reducing neurogenesis and neuronal plasticity [21]. Therefore, we examined the protein levels of NF-κB p65, MAPK p38 and proinflammatory cytokines in the hippocampus 6 h post-surgery and on POD 1 and POD 7. The surgery upregulated p-p65 and p-p38, whereas acetate significantly downregulated these proteins both 6 h post-surgery and on POD 1; however, no significant differences were found on POD 7 (Figure 3).

In addition, inflammation is known to increase the production of ROS and RNS, which further exacerbate inflammatory states. Thus, we measured the expression of NOX2 and inducible nitric oxide synthase (iNOS) through Western blot analysis. Surgery increased NOX2 and iNOS expression, while acetate inhibited surgery-induced oxidative stress by reducing the levels of these proteins on both POD 1 and POD 7 (Figure 3).

**Figure 4:**
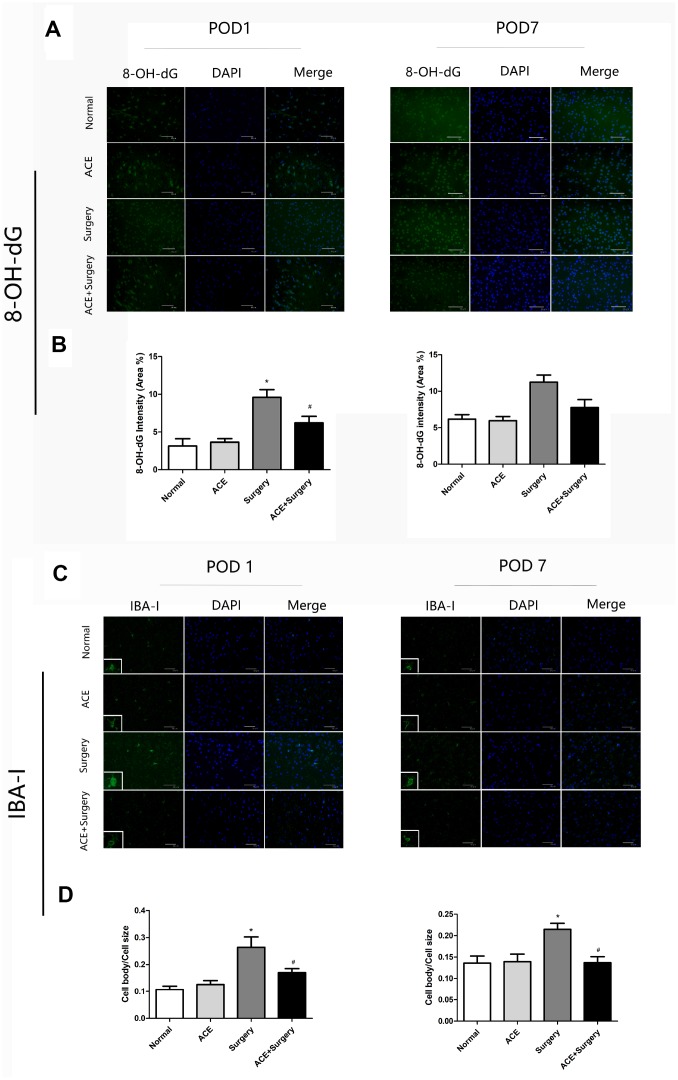
**Surgery-induced ROS overproduction and IBA-1 activation in the hippocampus were suppressed by acetate administration (n=5).** (**A**) Green fluorescence indicates 8-OH-dG levels on POD 1 and POD 7. (**B**) Bar graphs display the quantification of 8-OH-dG in the hippocampus. (**C**) Representative images of IBA-1 expression on POD 1 and POD 7. (**D**) Bar graphs indicate the cell body/cell size of IBA-1-labeled microglia in the four groups. Data are expressed as the mean±SEM, *P<0.05 vs. the normal and acetate groups, #P<0.05 vs. the surgery group. ACE: acetate. Scale bar = 50 μm.

Surgery-induced ROS generation was also measured by 8-hydroxy-20-deoxyguanosine (8-OH-dG) immunostaining, which detects oxidized nucleic acids resulting from cellular ROS damage [22] and has been used as a marker of DNA oxidation. The data revealed that surgery induced ROS overproduction in the hippocampus, while acetate treatment clearly attenuated ROS production on POD 1. Thus, acetate may function as an antioxidant. However, there were no significant differences on POD 7 (Figure 4A and 4B).

### Acetate treatment inhibited the activation of microglia in the hippocampus

Microglia may contribute to neuroinflammation and oxidative stress in PNDs. We measured ionized calcium-binding adaptor molecule 1 (IBA-1), a marker of microglial activation, through immunofluorescence analyses on POD 1 and POD 7. Then, we quantified the ratio of the microglial cell body to cell size as a measure of microglial activation. The cell body/cell size of IBA-1-labeled microglia was significantly greater in the surgery group than in the normal group on POD 1 and POD 7. Acetate partly reversed these alterations (Figure 4C and 4D), indicating that acetate may inhibit microglial activation and further alleviate the inflammatory response.

### Acetate exerted anti-inflammatory effects in BV2 cells

**Figure 5:**
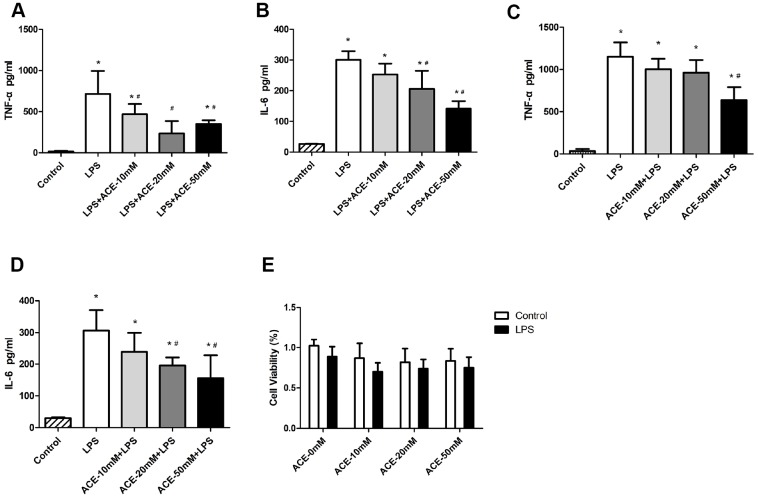
**The anti-inflammatory effects of acetate in BV2 cells (n=3 independent experiments).** During the acetate post-treatment, different concentrations of acetate (10, 20 or 50 mM) were added to the wells 30 min after LPS (100 ng/mL) stimulation. Inflammatory proteins were detected by ELISA 6 h after LPS stimulation. Bar graphs display the protein levels of TNF-α (**A**) and IL-6 (**B**). During the acetate pretreatment, acetate was added to the wells 30 min before LPS (100 ng/mL) stimulation, and inflammatory proteins were detected by ELISA as previously mentioned. Bar graphs display the protein levels of TNF-α (**C**) and IL-6 (**D**). (**E**) The MTT experiment revealed no significant differences in cell viability among the groups. Data are expressed as the mean±SEM, *P<0.05 vs. the control and acetate groups, #P<0.05 vs. the LPS group. ACE: acetate.

Since acetate exhibited neuroprotective effects by preventing PNDs in mice, we also evaluated the function of acetate *in vitro*. We used a neuroinflammation model in which BV2 cells were stimulated with LPS (100 ng/mL), as this is a common approach in PND research [23–25]. We treated the cells with various concentrations (10, 20 and 50 mM) of acetate before or after LPS treatment to determine the proper conditions for the *in vitro* experiments. When acetate was administered after LPS stimulation, 20 mM acetate effectively reduced TNF-α and IL-6 protein levels (Figure 5A and 5B); however, when acetate was administered before LPS stimulation, 50 mM acetate was required as the effective concentration (Figure 5C and 5D). These findings suggested that acetate was more effective as a post-treatment in BV2 cells. Therefore, post-treatment with 20 mM acetate was chosen for the *in vitro* experiments.

We also performed a 3-(4,5-dimethylthiazol-2-yl)-2,5-diphenyltetrazolium bromide (MTT) experiment, which indicated that the three tested concentrations of acetate did not alter cell viability (Figure 5E). Thus, the decreases in inflammatory protein levels were not due to increases in cell death. These data indicated that acetate also exerted anti-inflammatory effects in BV2 cells.

### Acetate suppressed inflammatory signaling pathways and oxidative stress in BV2 cells

**Figure 6:**
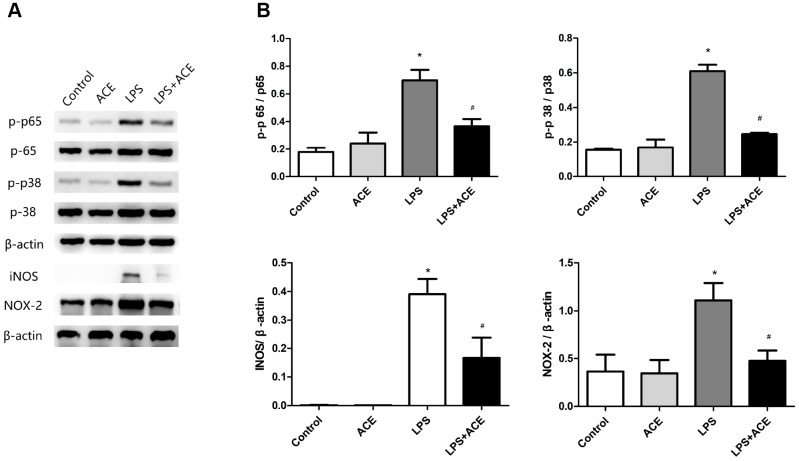
**Changes in inflammatory signaling pathways and oxidative stress markers in BV2 cells (n=5).** (**A**) Representative bands display the protein levels of p-p65, p-p38, iNOS and NOX2 in BV2 cells. The levels of p-p65 and p-p38 were detected 1 h after LPS stimulation, while the levels of iNOS and NOX2 were examined 24 h after LPS stimulation. (**B**) Bar graphs depict the quantification of p-p65/p65, p-p38/p38, iNOS/β-actin and NOX2/β-actin in BV2 cells. Data are expressed as the mean±SEM, *P<0.05 vs. the control and acetate groups, #P<0.05 vs. the LPS group. ACE: acetate.

We also measured the protein expression of NF-κB p65 and MAPK p38 *in vitro*. Acetate significantly attenuated the expression of p-p65 and p-p38 1 h after LPS stimulation. In addition, acetate reduced NOX2 and iNOS levels 24 h after LPS stimulation (Figure 6). These results demonstrated that acetate exerted similar protective effects in both BV2 cells and the PND model by attenuating the inflammatory response.

### The anti-inflammatory effects of acetate depended on GPR43 expression

**Figure 7:**
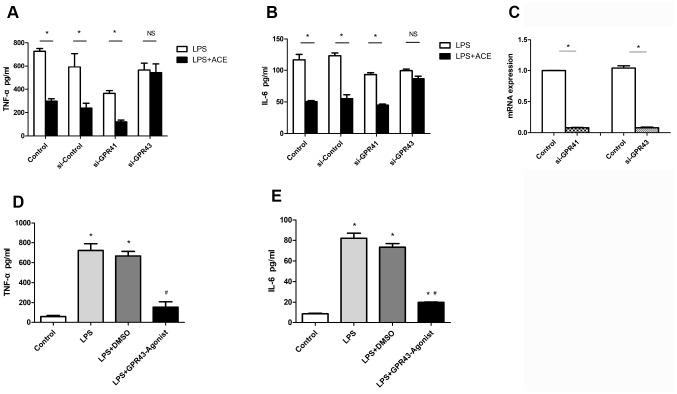
**The protective effects of acetate depended on GPR43 expression (n=3 independent experiments).** After being treated with si-GPR41 or si-GPR43 for 48 h, BV2 cells were stimulated for 6 h with LPS, with or without subsequent acetate treatment, and the supernatants were examined by ELISA. (**A**) Bar graph depicts the expression of TNF-α in the different groups. (**B**) Bar graph displays the protein levels of IL-6 in the four groups. (**C**) The interference efficiencies of si-GPR41 and si-GPR43 were assessed based on the respective mRNA levels in BV2 cells. (**D**) Bar graph displays the expression of TNF-α in cells treated with the GPR43 agonist (10 μM). (**E**) Bar graph displays the protein levels of IL-6 in cells treated with the GPR43 agonist. Data are expressed as the mean±SEM, *P<0.05 vs. the control group, #P<0.05 vs. the LPS and LPS+DMSO groups. DMSO was used as a solvent control. ACE: acetate.

Acetate has been confirmed to reduce inflammation and alter the immune response in various models [20, 26]. Acetate seems to influence immune cells mainly through G protein-coupled receptors (GPCRs) [27], and has exhibited similar affinities for GPR41 and GPR43 [28]. To evaluate whether acetate exerted neuroprotective effects by binding to GPCRs in microglia, we used small interfering RNAs (siRNAs) to knock down GPR41 and GPR43. The knockdown of GPR43 attenuated the protective effects of acetate in LPS-treated BV2 cells, whereas the knockdown of GPR41 did not (Figure 7A and 7B), suggesting that acetate probably inhibited neuroinflammation by activating GPR43. The interference efficiencies of si-GPR41 and si-GPR43 are presented in Figure 7C.

We also used a specific agonist of GPR43 to confirm that GPR43 could suppress the secretion of inflammatory proteins into the supernatants of LPS-treated BV2 cells. The GPR43 agonist had an inhibitory effect similar to that of acetate (Figure 7D and 7E). These results confirmed the correlation between acetate activity and GPR43 activation.

## DISCUSSION

In the present study, we explored the correlation between acetate treatment and neurocognitive deficits in a mouse model of PNDs. We discovered that acetate administration reduced the neuroinflammation and hippocampus-dependent memory impairment resulting from surgical trauma. Acetate effectively inhibited microglial activation, thus suppressing the inflammatory response, reducing oxidative stress and improving neurocognitive function. The effects of acetate seemed to depend on its ability to activate GPR43.

Surgery-induced PNDs are relatively prevalent, occurring in 10-54% of patients during the first few weeks post-surgery [29]. However, postoperative neurocognitive complications are not simply confined to the acute post-surgery phase; they may lead to chronic cognitive deficits and an increased risk of mortality, creating a tremendous burden for families and society [30]. According to previous studies, various factors influence the occurrence of PNDs, such as gender, education level, age, etc. Among these factors, advanced age is an independent risk factor frequently observed in clinical studies [31]. The normal aging process in the brain predisposes aged individuals to develop neurocognitive disorders. Aging inhibits the physiologic functions of many organ systems, including the brain, and thus increases the vulnerability of patients to systemic stressors such as surgery [32]. Therefore, aged mice were used to simulate the clinical status of patients with PNDs in this study.

Exploratory laparotomy with isoflurane anesthesia is a widespread method of inducing PNDs in aged mice [22, 23, 33], and closely replicates clinical scenarios; thus, we used this method to generate our PND animal model. Our OFT and MWM test results demonstrated that the exploratory laparotomy induced significant neurocognitive deficits. Isoflurane alone has also been found to induce cognitive deficits in various studies [34, 35]. The neurotoxic mechanisms of isoflurane include altering calcium homeostasis [36], increasing ROS production and inducing neuroinflammation [37]. Our results provided partial evidence that isoflurane evoked cognitive impairment on POD 7 (Supplementary Figure 1).

Microglia are important contributors to neuroinflammation, a common feature of PNDs. Surgery-induced peripheral inflammation may activate the otherwise silent microglia, promoting the release of inflammatory cytokines into the CNS and thereby inducing neuroinflammation. The inhibition of microglia has been reported to reduce the levels of proinflammatory cytokines such as IL-1β and TNF-α, thus inhibiting neuroinflammation and enhancing neurocognitive function [9]. The present study demonstrated that acetate can suppress neuroinflammation in the hippocampus. Acetate treatment not only reduced the peripheral expression of inflammatory factors (TNF-α, IL-6 and IL-1β), but also downregulated these proteins and the associated signaling pathways (NF-κB and p38-MAPK)in the hippocampus 6 h after surgery. This time point was selected because these proinflammatory cytokines were highly expressed in both the plasma and the hippocampus 6 h after surgery [24].

Surgery can trigger acute systemic inflammation, followed by neuroinflammation and synaptic dysfunction, which can lead to cognitive deficits; thus, the inhibition of systemic inflammation via various treatments has been reported to prevent neuroinflammation and neurocognitive changes [38]. Our data demonstrated that acetate treatment could suppress both systemic inflammation and neuroinflammation. In fact, acetate may exert functions in both the periphery and the CNS because it can be absorbed by several mechanisms, including passive diffusion, active membrane transport and GPCR-dependent uptake. Therefore, the effects of acetate on PNDs are probably comprehensive.

Surgical trauma also induced inflammation-regulating pathways in the present study. After surgery, p-p65, p-p38 and cytokines were clearly upregulated in the hippocampus, while acetate administration inhibited these changes both 6 h post-surgery and on POD 1. These data were consistent with the results of previous studies by Wang et al., who found that blocking the NF-κB or p-38 MAPK pathway alleviated CNS inflammation and neurocognitive deficits [8, 39]. However, p-p65, p-p38 and proinflammatory cytokine levels were not significantly altered on POD 7, probably because the associated inflammatory signaling pathways were mainly induced during the acute inflammation phase and returned to the normal state in the chronic phase.

The activation of microglia may promote the release of ROS and RNS, which can modify lipids, proteins and nucleic acids [13]. Due to the high metabolic rate and low antioxidant level of the CNS, neurons are prone to damage from these reactive species [40]. NADPH oxidase is an important source of ROS in phagocytes, including microglia [41]. Various isoforms in the NADPH oxidase family regulate the microglial phenotype and subsequent neuroinflammation. Among these isoforms, NOX2 exerts vital functions in the CNS [42]. INOS has been reported to be a marker of the classical activation phenotype of microglia (M1), and may promote the development of neuroinflammation and neurotoxicity [43]. Acetate suppressed the surgery-induced upregulation of these proteins in our PND model, illustrating its antioxidant effects. We obtained similar results *in vitro*, as acetate exerted anti-inflammatory and antioxidant effects in LPS-stimulated BV2 cells.

SCFAs are abundant metabolites in the intestinal tract. Acetate, propionate and butyrate are the most extensively detected SCFAs in the intestinal tract, and are present at a molar ratio of 60:20:20, respectively [44]. It is worth noting that SCFAs are not limited to the intestinal tract, but can diffuse systemically and be detected in the blood. In the present study, we selected acetate over other SCFAs because acetate can reach concentrations of 100-150 μM or higher in the circulating blood and thus can impact peripheral tissues [45], whereas propionate and butyrate circulate at markedly lower levels. *In vivo* acetate treatments have been described in several previous studies [19, 20, 46]. In mice, acetate can reach concentrations of 15 mmol/L and 1-2 mmol/L in the intestines and blood, respectively. Acetate regulates various physiological functions, including inflammation and immune system activity. In a gastric mucosal injury model, acetate significantly inhibited TNF-α, IL-6 and NF-κB p65 expression, increased glutathione levels and enhanced superoxide dismutase activity, demonstrating its anti-inflammatory and antioxidant effects [26]. However, acetate has also been reported to promote inflammation [19], indicating that it may have different effects in different models [47, 48].

Acetate can be absorbed by three pathways in the intestinal tract: passive diffusion (mainly in its nonionized form), active membrane transport (through Slc16a1 and Slc5a8) and GPCR-dependent transport [49]. Acetate can elicit its effects by activating GPCRs, suppressing histone deacetylases or stimulating histone acetyltransferases. In this study, we investigated the relationship between acetate and GPCRs (GPR41 and GPR43) because of the important influence of GPCR-associated pathways on immune cells [49]. We hypothesized that the activity of microglia, the innate immunocytes of the CNS, might be inhibited by GPCRs on the cell membrane. Since acetate has been reported to activate both GPR43 and GPR41 [50], we used siRNA to silence each of these proteins, and found that the effects of acetate mainly depended on GPR43 expression in BV2 cells. Given the above findings, acetate in the intestinal tract most likely enters the bloodstream through passive diffusion or transport, and then circulates to the CNS, where it activates GPR43 on microglia. This activation of GPR43 may suppress microglial activity, thereby inhibiting neuroinflammation, reducing oxidative stress and improving neurocognitive function.

This study had several limitations. First, we did not measure the concentration of acetate in the CNS. Second, the use of GPR43- or GPR41-deficient mice would have provided stronger evidence for the protective mechanism of acetate. We will consider using genetic approaches in future studies. In addition, further research is needed to determine how acetate alters GPCR signal transduction in the PND model. In future studies, we will investigate whether acetate exerts neuroprotective effects in the PND model by activating histone acetyltransferases.

In summary, surgery activated hippocampal microglia in aged mice, resulting in neuroinflammation and hippocampus-dependent neurocognitive impairment. By activating GPR43, acetate inhibited microglial activity and reduced neuroinflammation and oxidative stress, thereby improving neurocognition.

## MATERIALS AND METHODS

### Animals

C57BL/6 mice (12 months old, 25-35 g) were purchased from the experimental animal care center of Navy Medical University (Shanghai, China) and were housed under controlled conditions (20 ± 2 °C and 50 ± 10% humidity, 12 h light/dark cycle) with free access to food and water. All experiments were conducted in strict accordance with protocols approved by the local ethics committee for animal research at Changhai Hospital, and conformed to the Animal Research Guidelines. All experiments were conducted after seven days of acclimatization.

### Surgery

Mice were anesthetized with 2-3% isoflurane and oxygen at 2 L/min in an induction chamber for 30 min. Then, a modified abdominal exploratory surgery was performed with 1.5% isoflurane [51]. Briefly, after the abdominal region was shaved and cleaned, a 1.5-cm median abdominal incision was made, and the peritoneal cavity was penetrated. Then, the viscera (liver, spleen and kidneys) and intestines were explored, and approximately 10 cm of the small intestine was exteriorized from the peritoneal cavity, covered with moist gauze and manipulated by hand. Finally, the peritoneal cavity was sutured layer by layer with sterile 4-0 chromic gut sutures. The entire surgical procedure lasted 30 min. The mice in the control groups underwent neither anesthesia nor surgery.

### Open-field test (OFT)

OFTs were performed in plastic chambers consisting of four boxes (50 cm × 50 cm × 50 cm) to evaluate the locomotor activity of the experimental mice. Each mouse was gently placed in the center of the chamber, and the movement trajectory was automatically recorded for 5 min with a video tracking system (Supermaze+ V 3.0 software, Xinruan Information Technology Co., Ltd., Shanghai, China). The total distance and pause time were recorded and analyzed. All OFTs were carried out on POD 3.

### Morris water maze (MWM)

The MWM consisted of two parts: the preoperative training phase and the postoperative testing phase. The water maze tank was 120 cm in diameter and 30 cm in depth, and was filled with water at 20 °C. The water maze was divided into four quadrants. The hidden platform was located in the middle of one quadrant, which was defined as the target quadrant. Each mouse was placed in one of the quadrants and allowed 60 s to locate the hidden platform. If the mouse failed to find the platform within 60 s, it was guided to the platform and allowed to stay on it for 15 s. Then, it was dried in a warm environment and placed in its cage. Four trials were carried out on each mouse, each starting from a different quadrant. Preoperative training was conducted for five consecutive days before surgery. During the testing phase, the platform was removed, and Supermaze+ V 3.0 software (Xinruan Information Technology Co., Ltd.) was used to record the trajectory of the mouse automatically for 60 s. Testing was conducted on POD 3 and POD 7.

### Drugs and treatments

The mice were divided into four groups: a normal group, an acetate group, a surgery group and an acetate + surgery group. The mice in the acetate and acetate + surgery groups were given 200 mM acetate (acetate solution, pH 5.2, Sigma-Aldrich, lot 3863) in their drinking water for seven consecutive days before surgery. The dose (200 mM) of acetate *in vivo* was chosen based on previous studies [19, 20]. To evaluate the acute inflammatory response, we obtained hippocampal tissues and peripheral blood (by heart puncture) 6 h after surgery. Hippocampal tissues were also harvested on POD 1 and POD 7 for Western blot and immunofluorescence analyses.

### BV2 cells

The murine microglial cell line BV2 was used in this study. The cells were kept in a 5% CO_2_ incubator and were maintained in Dulbecco’s modified Eagle’s medium (DMEM; HyClone Co., USA) with 10% fetal bovine serum (FBS; HyClone Co.), 100 U/mL penicillin and 100 μg/mL streptomycin at 37 °C. The cells were treated with LPS (100 ng/mL, *Escherichia coli* O111:B4, Sigma, China) with or without acetate.

BV2 cells were cultured in 24-well plates for the enzyme-linked immunosorbent assay (ELISA) and in six-well plates for Western blot analysis. The levels of inflammatory proteins were detected by ELISA after the cells had been treated with LPS for 6 h. The protein levels of signaling molecules (p-p38 and p-p65) and oxidative stress factors (iNOS and NOX2) were assessed by Western blotting 1 h and 24 h after LPS treatment. For all *in vitro* experiments, acetate treatment started 30 min before or after LPS stimulation, so the cells were ultimately treated with acetate for 6.5 h or 5.5 h, respectively.

### ELISA

The concentrations of TNF-α, IL-6 and IL-1β in the hippocampus, peripheral blood and cell supernatants were detected with ELISA kits (Thermo, USA). Hippocampal samples were homogenized and centrifuged at 12,000 × *g* for 10 min. The supernatants were collected, and the proteins therein were quantified with a bicinchoninic acid assay. Similarly, peripheral blood was centrifuged as described above, and the plasma was obtained. BV2 cells were seeded in 24-well plates at a density of 1×10^5^ cells per well for 6 h. The cells were then centrifuged at 12,000 × *g* for 10 min, and the supernatants were collected. The ELISA experiments were performed in accordance with the kit manufacturer’s instructions. The absorbance was read at a wavelength of 450 nm with a spectrophotometer.

### Cell viability detection

Cell viability was evaluated with an MTT assay kit (Comiike Biotechnology Co., China) in accordance with the manufacturer’s instructions. Cells were treated with LPS for 6 h, with or without different concentrations of acetate, and then the culture medium was aspirated. MTT (0.5 mg/mL) was added to the cells, and the cells were incubated at 37 °C for 4 h. Finally, dissolution liquid was added to each well to dissolve the formazan crystals. The absorbance was read with a spectrophotometer at 570 nm.

### Western blot analysis

Proteins in hippocampal tissues and cell culture media were extracted with a radioimmunoprecipitation assay lysis buffer (Beyotime, China) containing a protease inhibitor cocktail, and the samples were centrifuged at 12,000 × *g* for 10 min at 4 °C. The protein concentrations were detected with a bicinchoninic acid assay (Thermo, China). The denatured proteins were separated by sodium dodecyl sulfate polyacrylamide gel electrophoresis and then transferred onto polyvinylidene difluoride membranes (Merck, Germany), which were blocked with 5% skim milk in Tris-buffered saline with Tween. Then, the membranes were incubated at 4 °C overnight with primary antibodies against p-p65 (1:1000, CST, USA), p-65 (1:1000, CST), p-p38 (1:1000, CST), p-38 (1:1000, CST), NOX2 (1:4000, Abcam, USA), iNOS (1:1000, Abcam) or beta-actin (1:2000, Sigma, USA). After being washed in Tris-buffered saline with Tween, the membranes were incubated with horseradish peroxidase-conjugated secondary antibodies (1:2000, CST) at room temperature for 2 h, and the specific bands were detected with an enhanced chemiluminescence kit (Pierce, USA). ImageJ software (National Institutes of Health, Bethesda, MD, USA) was used to analyze the results.

### Immunofluorescence

Mice were sacrificed, and their brains were post-fixed in 4% paraformaldehyde, dehydrated with 30% sucrose at 4 °C overnight and then embedded in paraffin. Sections were cut to a thickness of 10 μm on glass slides. The sections were blocked with 10% donkey serum for 1 h, incubated with mouse anti-IBA-1 (Proteintech, USA) or 8-OH-dG (Abcam) antibodies overnight at 4 °C, and incubated with secondary antibodies for 1 h at room temperature. After being washed, the sections were incubated with 4’,6-diamidino-2-phenylindole for nuclear staining.

Three fields and five sections were used in this experiment, and the CA1 region of the hippocampus was analyzed. The area of selected cells was converted into a binary image. Total immunoreactivity was calculated as the percentage area density, defined as the positively stained area divided by the sum of the positively and negatively stained areas in the image field. In the IBA-1 analysis, microglial activation was expressed as the cell body/cell size of IBA-1-stained microglia [52]. The pictures are shown at 400× magnification. Images were captured with a microscope and analyzed with ImageJ software (National Institutes of Health).

### siRNA interference and GPR43 agonist

BV2 cells were cultured in half of the culture volume of FBS-free DMEM and transfected for 6 h with 3 ng/mL of siRNA (si-GPR41 or si-GPR43) or control siRNA (GenePharma, China). INTERFERin (Invitrogen, USA) was used for the transfection in accordance with the manufacturer’s instructions. The other half of the complete culture medium was added 6 h later, and the cells were cultured for 48 h. After 48 h of interference, the cells were used for further experiments. The sequences of the siRNAs were as follows: si-GPR41, GCUUCUUUCUUGGCAAUUAdTdT; si-GPR43, GC UGGUACCUACCAAAGAUdTdT.

A specific GPR43 agonist was used in our study. Thirty minutes after the LPS stimulation, the GPR43 agonist (10 μM in dimethyl sulfoxide [DMSO]) and DMSO (0.2%) were added to the culture medium. After 6 h of LPS stimulation, the supernatant was collected, and TNF-α and IL-6 levels were detected via ELISA.

### Statistical analysis

The data are shown as the mean ± standard error of the mean (SEM). Two‒way analysis of variance (ANOVA) was used to assess latency in the MWM test. Differences between two groups were assessed by student's t test. The Shapiro-Wilk normality test was used to detect whether the data were normally distributed. If the data were normally distributed, the groups were compared by ANOVA followed by the Student‒Newman‒Keuls post-hoc test. If the data were not normally distributed, a non-parametric test (the Kruskal-Wallis test) was selected. Statistical analyses were performed with GraphPad Prism 6 (GraphPad Software, Inc., La Jolla, CA, USA). P values <0.05 were considered statistically significant.

## Supplementary Material

Supplementary Figure 1
