# Supplementary material for: Acetate attenuates perioperative neurocognitive disorders in aged mice
Source: Aging (Albany NY). 2020 Feb 26;12(4):3862–79. doi: 10.18632/aging.102856 (PMC7066918; doi:10.18632/aging.102856)
Supplement: Supplementary Figure 1 [file aging-12-102856-s001..pdf]

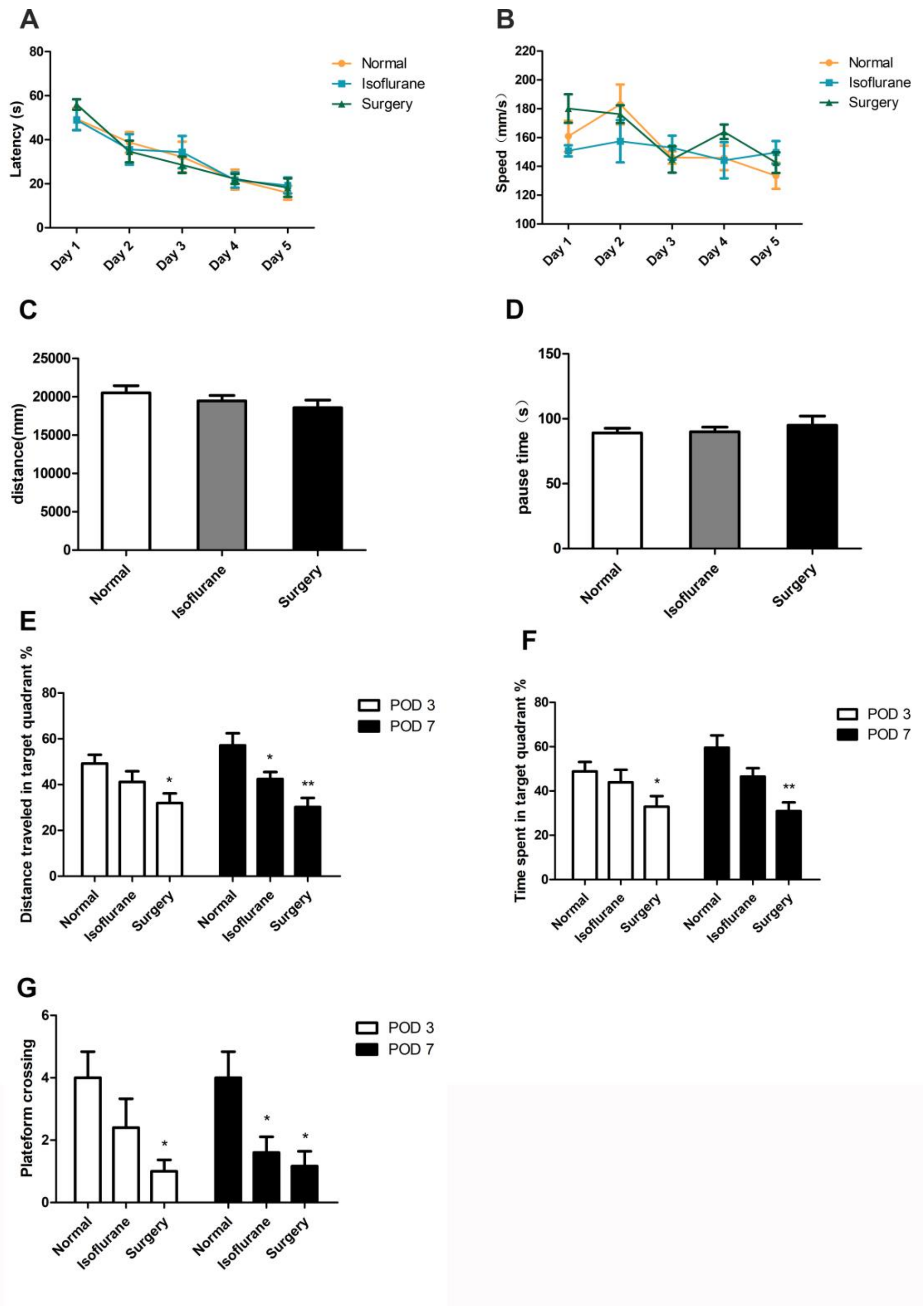

Supplementary Figure 1. The neurocognitive changes evoked by surgery and isoflurane in different groups (n=8). The latency (**A**) and average speed (**B**) of the training phase showed all mice successfully found the hidden platform at the fifth day. There were no significant differences between the three groups in open field test including total distance (**C**) and pause time (**D**) 3 days after surgery, P>0.05. The percentage of distance traveled in target quadrant (**E**), time spent in target quadrant (**F**), and platform crossings (**G**) both on POD 3 and POD 7 were showed in the figure. Data are expressed as mean±SEM, the ANOVA was used for the statistics, *P<0.05 vs. the normal group, **P<0.01 vs. the normal group.
